# Association between grip strength and stress urinary incontinence of NHANES 2011–2014

**DOI:** 10.1186/s12905-023-02628-1

**Published:** 2023-10-03

**Authors:** Nieke Zhang, Weipu Mao, Si Sun, Guanyuan Zhang, Naipeng Shi, Chi Yao, Ning Liu, Shuqiu Chen, Wei Gao, Lei Zhang, Ming Chen, Xiangyu Zou

**Affiliations:** 1 Department of Urology, Zhongda Hospital, Southeast University, Nanjing, China; 2 Institute of Urology, Medical School, Southeast University, Nanjing, China; 3 School of Basic Medical Sciences, Weifang Medical University, Weifang, China; 4 Department of Urology, Shanghai Children’s Medical Center, Shanghai Jiao Tong University School of Medicine, Shanghai, China

**Keywords:** Grip strength, Relative grip strength, Stress urinary incontinence, NHANES, PSM

## Abstract

**Objectives:**

To investigate the association between grip strength (GS) and relative grip strength (rGS) with the prevalence and severity risk of SUI.

**Methods:**

Female patients were retrieved from the NHANES 2011–2014. GS was measured using a digital hand dynamometer, rGS was defined as grip strength divided by BMI. Samples were classified into four groups based on quartiles of GS and rGS distribution (Q1-Q4)。Logistic regression models were established to detect the relationship between GS or rGS and SUI. The potential bias of baseline variables between SUI and non-SUI groups was controlled by performing the propensity score matching (PSM).

**Results:**

A total of 4263 samples were included, with 3085 (85%) people in non-SUI group and 1178 (27.6%) people in SUI group. GS and rGS levels of people without SUI were higher than that of SUI patients. Monthly SUI patients’ GS and rGS levels were higher than weekly SUI patients’ level. Logistic regression analysis showed that risks of prevalence and severity of SUI decreased with increasing levels of GS and rGS. rGS was found to have a stronger association with SUI than GS [prevalence: GS: Q4 vs. Q1: aOR = 0.633, 95%CI = 0.508–0.789, *p* < 0.001; rGS: Q4 vs. Q1: aOR = 0.365, 95%CI = 0.290–0.459, *p* < 0.001; severity: GS: Q4 vs. Q1: aOR = 0.727, 95%CI = 0.600–0.881, *p* = 0.001; rGS: Q4 vs. Q1: aOR = 0.371, 95%CI = 0.282–0.488, *p* < 0.001]. The results of PSM confirmed that GS and rGS were correlated with SUI.

**Conclusions:**

Lower levels of GS and rGS are associated with an increased prevalence and severity risk of SUI.

**Supplementary Information:**

The online version contains supplementary material available at 10.1186/s12905-023-02628-1.

## Introduction

Urinary incontinence (UI) is a pathological condition that has adverse effects on many people, with stress urinary incontinence (SUI) being a common subtype characterized by involuntary leakage during effort or exertion [1, 2]. SUI is more prevalent in women and is associated with bladder or pelvic floor muscle dysfunction [3]. The prevalence of SUI peaks among middle-aged women and declines gradually thereafter [4]. The pathological mechanism of SUI is related to age-associated muscle mass and nerve decline, as well as muscle dysfunction [5]. Synthetic mid-urethral slings are proposed as the gold standard treatment of SUI by the European Society of Urology and the European Urogynaecological Association (EUA) [6].

Grip strength, which is an indicator of total muscle strength [7], declines gradually after middle age [8], and low grip strength is associated with adverse outcomes, such as disability, longer hospital stays, and mortality [9, 10]. Additionally, lower grip strength levels are associated with a higher morbidity of various diseases, including cancer, cardiovascular diseases, respiratory diseases, and sarcopenia [11, 12].

Several studies have explored the relationship between grip strength and SUI [13, 14], but the results are still controversial, and potential confounding factors have not been adequately controlled. Therefore, the purpose of this study is to investigate the association between grip strength and SUI by examining the data extracted from the NHANES 2011–2014. The study aims to determine whether grip strength has an impact on the prevalence and severity risk of SUI.

## Methods

### Study design and statistical analysis

This study is a retrospective analysis. The grip strength was measured using a handheld dynamometer, and the SUI was assessed through a self-reported questionnaire. The degree of SUI was classified into three categories: no SUI, monthly SUI, and weekly SUI. The grip strength was categorized into quartiles based on gender and body mass index (BMI). The other demographic and clinical characteristics, including education levels, BMI, age, marital status, hypertension, diabetes mellitus, and renal function status, were also collected.

Statistical analysis was conducted using the SAS software (version 9.4; SAS Institute, Cary, NC, USA). The descriptive analysis was performed to examine the demographic and clinical characteristics of the study population. The prevalence of SUI was calculated and compared between groups based on grip strength and other characteristics. The association between grip strength and SUI was examined using logistic regression models. In the regression analysis, the grip strength was modeled both as a continuous variable and as a categorical variable (quartiles). Covariates were adjusted for in the regression models, including age, education level, marital status, hypertension, diabetes mellitus, BMI, and renal function status. The odds ratios (ORs) and 95% confidence intervals (CIs) were calculated. A *p* value < 0.05 was considered significant statistically.

### Grip strength measurement

A digital hand dynamometer manufactured by Lafayette Instrument Company (USA) was utilized to measure grip strength in this study. Samples were instructed to stand with both arms extended fully at their sides, without touching their bodies. They were then asked to squeeze the dynamometer with as much force as possible for less than three seconds, alternating each hand three times. A rest interval of at least 30 s was implemented between each trial. The minimum measurement unit was 0.1 kg with an accuracy of ± 2.0 kg. To ensure consistency, the maximum score from six grip strength measurements was used for statistical analysis [9].

Previous research has suggested that relative grip strength (rGS), defined as grip strength divided by body mass index, is more strongly associated with cardiometabolic disease biomarkers than absolute grip strength [15]. However, for predicting cancer outcomes, absolute grip strength has been found to be more effective than rGS [16]. Therefore, both rGS and absolute grip strength were used to assess samples’ muscle strength in this study.

### Study variables

The study variables were obtained from the NHANES database, including education levels, race, BMI, age, hypertension, diabetes mellitus, marital status, smoking status, physical activity status, grip strength, blood urea nitrogen, creatinine, and uric acid. The clinical characteristics of the patients were categorized based on the following variables: education (less than high school, high school or equivalent, college or above),race (non-Hispanic white, non-Hispanic black, Mexican American, other Hispanic, and other), body mass index (normal, overweight, or obese), age (less than 40 and 40 years and older), hypertension (yes or no), diabetes mellitus (yes, no, or borderline), marital status (married and unmarried), and smoking status (never, former, or current). Physical activity status was classified as either vigorous (yes or no) or moderate (yes or no). The mean grip strength and relative grip strength (rGS) were computed for each person.

### Statistical analysis

To analyze the associations between study variables, various statistical tests were conducted. The chi-square test was used to analyze categorical variables, and the t-test for slope was used in generalized linear models for continuous variables. Continuous variables were presented as means with standard deviation (SD), and categorical variables were expressed as proportions. Samples were classified into four groups based on quartiles of GS and rGS distribution (Q1-Q4), and the effect of included variables on rGS was evaluated. Propensity score matching (PSM) was performed in a 1:1 ratio to control for potential bias in baseline variables across different groups, including age, race, marital status, education, BMI, diabetes mellitus, smoking status, physical activity status, blood urea nitrogen, creatinine and uric acid.

Linear and logistic regression models were used to evaluate the association of GS or rGS with the risk of stress urinary incontinence (SUI) and its severity before and after PSM. Adjusted odds ratios (aOR) and 95% confidence intervals (CI) were calculated for the prevalence and severity of SUI related to quartiles of GS and rGS. Five logistic regression models were used for the analyses, starting with the univariate analysis (model 0) and subsequently adjusting for age and BMI (model 1), race, education, and marital status (model 2), hypertension and diabetes mellitus (model 3), and smoking status, physical activity status, blood urea nitrogen, creatinine, and uric acid (model 4).

Statistical analyses were performed using the Statistical Package for the Social Science software (version 26.0; SPSS, Chicago, IL, USA) and R software (version 4.1.0, http://www.r-project.org/). A *p*-value of less than 0.05 was considered statistically significant.

## Results

### Samples’ general characteristics

**Fig. 1:**
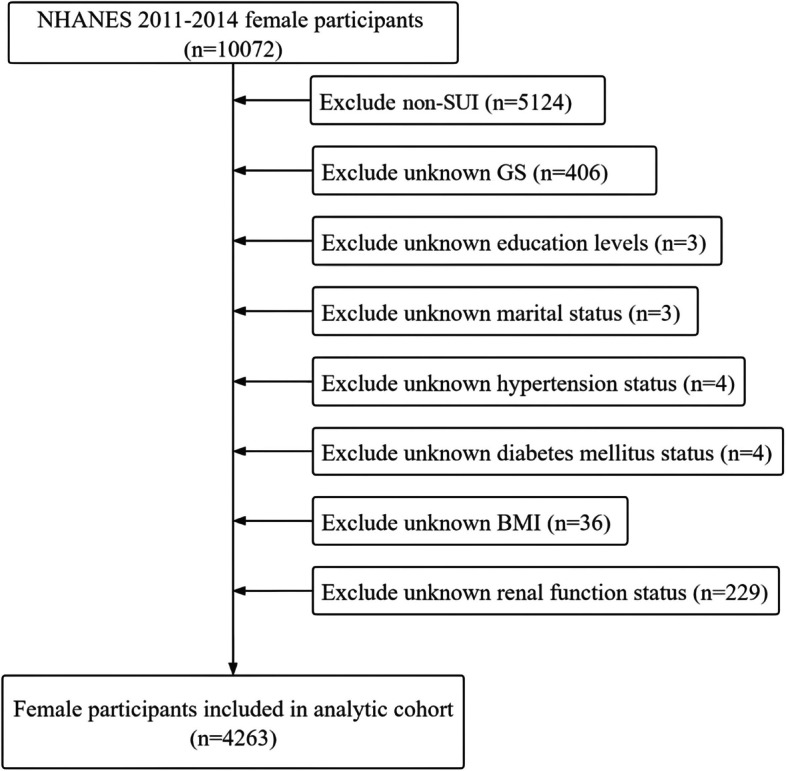
Schematic flow diagram of inclusion and exclusion criteria for our study cohort

**Fig. 2:**
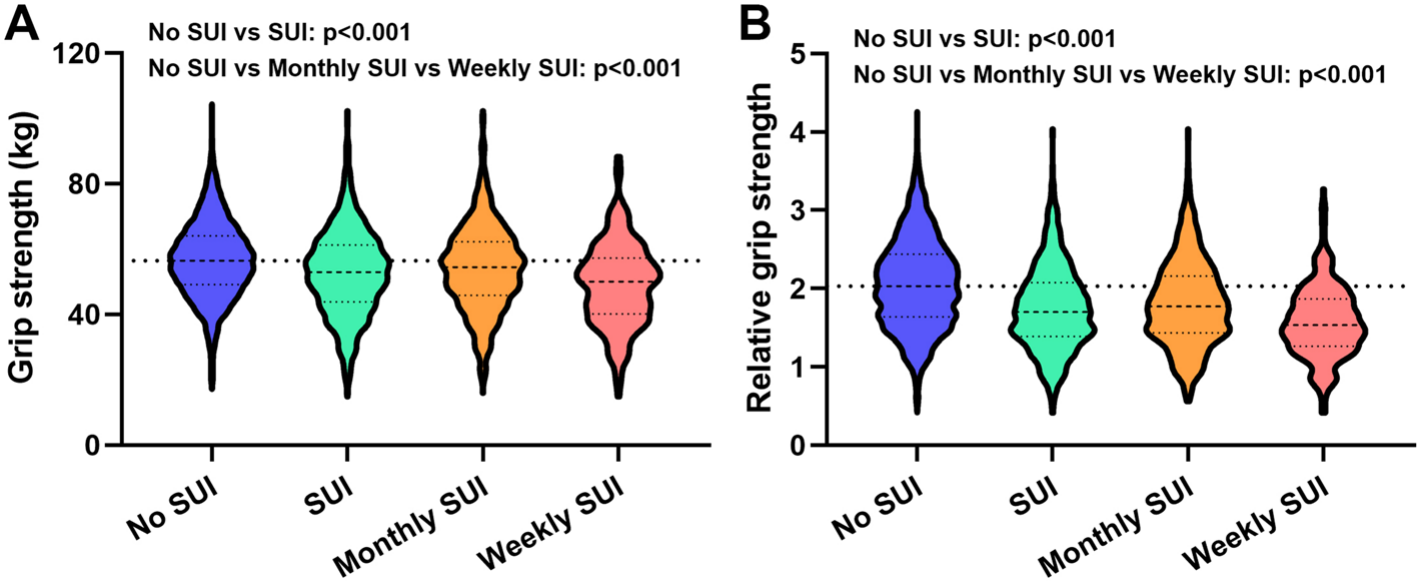
GS and rGS levels of no SUI, SUI, monthly SUI and weekly SUI patients before PSM

As shown in Fig. 1, after a rigorous selection process, a total of 4263 samples were included in this study from the NHANES database (2011–2014). Of these, 3085 (85%) did not have SUI, while 1178 (27.6%) had SUI. Among the SUI patients, 870 (20.4%) experienced monthly SUI and 308 (7.2%) experienced weekly SUI. Table 1 displays the demographic and clinical characteristics of the samples before propensity score matching (PSM). The mean GS level of individuals without SUI was significantly higher (56.61, 11.56) than that of SUI patients (52.75, 12.99). Additionally, the mean GS level of monthly SUI patients (54.02, 12.88) was higher than that of weekly SUI patients (49.18, 12.66). Similar results were observed for rGS levels, with no SUI patients (2.06, 0.57) having higher levels than SUI patients (1.75, 0.54), and monthly SUI patients (1.81, 0.55) having higher levels than weekly SUI patients (1.57, 0.46). Figure 2 displays the GS and rGS levels before PSM. Chi-square tests revealed significant differences in age, race, marital status, education, body mass index, hypertension, diabetes mellitus, smoking status, and physical activity status between the no SUI and SUI groups, as well as between the no SUI, monthly SUI, and weekly SUI groups. Significant differences were also observed in blood urea nitrogen, creatinine, and uric acid levels among the different groups (all *p* < 0.001).

**Table 1:** Clinical characteristics of the patients according to the with or without and severity of SUI before PSM

**Characteristic**	**All patients**	**No SUI**	**SUI**			***P value***^**a**^	***P value***^**b**^
			**SUI**	**Monthly SUI**	**Weekly SUI**		
		**No. (%)**	**No. (%)**	**No. (%)**	**No. (%)**		
Total patients	4263	3085 (72.4)	1178 (27.6)	870 (20.4)	308 (7.2)		
Age, years						< 0.001	< 0.001
Mean, SD	48.9, 17.5	45.82, 16.91	56.88, 16.46	55.15, 16.44	61.8, 15.5	< 0.001	< 0.001
< 40	1465 (34.4)	1260 (40.8)	205 (17.4)	173 (19.9)	32 (10.4)		
≥ 40	2798 (65.6)	1825 (59.2)	973 (82.6)	697 (80.1)	276 (98.6)		
Race						< 0.001	< 0.001
Non-Hispanic white	1822 (42.7)	1276 (41.4)	546 (46.3)	401 (46.1)	145 (47.1)		
Non-Hispanic black	975 (22.9)	664 (21.5)	311 (26.4)	237 (27.2)	74 (24.0)		
Mexican American	468 (11.0)	352 (11.4)	116 (9.8)	85 (9.8)	31 (10.1)		
Other Hispanic	419 (9.8)	312 (10.1)	107 (9.1)	67 (7.7)	40 (13.0)		
Other	579 (13.6)	481 (15.6)	98 (8.)	80 (9.2)	18 (5.8)		
Marital status						< 0.001	< 0.001
Married	1961(46.0)	1488 (48.2)	473 (40.2)	369 (42.4)	104 (33.8)		
Unmarried	2302 (54.0)	1597 (51.8)	705 (59.8)	501 (57.6)	204 (66.2)		
Education						< 0.001	< 0.001
Less than high school	814 (19.1)	559 (18.1)	255 (21.6)	157 (18.0)	98 (31.8)		
High school or equivalent	872 (20.5)	595 (19.3)	277 (23.5)	211 (24.3)	66 (21.4)		
College or above	2577 (60.5)	1931 (62.6)	646 (54.8)	502 (57.7)	144 (46.8)		
Body mass index, kg/m^2^						< 0.001	< 0.001
Mean, SD	29.58, 7.71	28.81, 7.35	31.59, 8.25	31.25, 8.09	32.57, 8.62	< 0.001	< 0.001
Normal (< 25,0)	1321 (31.0)	1060 (34.4)	261 (22.2)	202 (23.2)	59 (19.2)		
Overweight (250–29.9)	1177 (27.6)	879 (28.5)	298 (25.3)	221 (25.4)	77 (25.0)		
Obese (≥ 30.0)	1765 (41.4)	1146 (37.1)	619 (52.5)	447 (51.4)	172 (55.8)		
Hypertension						< 0.001	< 0.001
No	1581 (37.1)	979 (31.7)	602 (51.1)	412 (47.4)	190 (61.7)		
Yes	2682 (62.9)	2106 (68.3)	576 (48.9)	458 (52.6)	118 (38.3)		
Diabetes mellitus						< 0.001	< 0.001
Yes	510 (12.0)	278 (9.0)	232 (19.7)	137 (15.7)	95 (30.8)		
No	3638 (85.3)	2745 (89.0)	893 (75.8)	693 (79.7)	200 (64.9)		
Borderline	115 (2.7)	62 (2.0)	53 (4.5)	40 (4.6)	13 (4.2)		
Smoking status						< 0.001	< 0.001
Never	2772 (65.0)	2065 (66.9)	707 (60.0)	534 (61.4)	173 (56.2)		
Former	781 (18.3)	515 (16.7)	266 (22.6)	190 (21.8)	76 (24.7)		
Current	710 (16.7)	505 (16.4)	205 (17.4)	146 (16.8)	59 (19.2)		
Physical activity status							
Vigorous						< 0.001	< 0.001
Yes	791 (18.6)	661 (21.4)	130 (11.0)	111 (12.8)	19 (6.2)		
No	3472 (81.4)	2424 (78.6)	1048 (89.0)	759 (87.2)	289 (93.8)		
Moderate						< 0.001	< 0.001
Yes	1842 (43.2)	1400 (45.4)	442 (37.5)	345 (39.7)	97 (31.5)		
No	2421 (56.8)	1685 (54.6)	736 (62.5)	525 (60.3)	211 (68.5)		
Grip strength (kg)							
Mean, SD	55.54, 12.09	56.61, 11.56	52.75, 12.99	54.02, 12.88	49.18, 12.66	< 0.001	< 0.001
Relative grip strength	1.97, 0.58	2.06, 0.57	1.75, 0.54	1.81, 0.55	1.57, 0.46	< 0.001	< 0.001
Blood urea nitrogen (mmol/L)	12.42, 5.81	11.99, 5.59	13.54, 6.19	13.06, 5.87	14.87, 6.88	< 0.001	< 0.001
Creatinine (mg/dL)	0.80, 0.43	0.78, 0.44	0.84, 0.39	0.81, 0.30	0.90, 0.58	< 0.001	< 0.001
Uric acid (mg/dL)	4.89, 1.31	4.79, 1.25	5.17, 1.42	5.12, 1.43	5.32, 1.40	< 0.001	< 0.001

*PSM* Propensity score matching, *SUI* Stress urinary incontinence

For categorical variables, *P* values were analyzed by chi-square tests. For continuous variables, the t-test for slope was used in generalized linear models

^a^Chi-square detected the difference between No SUI group and SUI group

^b^Chi-square detected the difference between No SUI group, Monthly SUI group and Weekly SUI group

### Identification of influence factors of rGS and GS before PSM

Table 2 shows that the differences of race, marital status, education, body mass index, hypertension, diabetes mellitus, smoking status, physical activity status, blood urea nitrogen, creatinine, uric acid between four groups are statistically significant (*p* = 0.001 of marital status, *p* < 0.001 of the other variables). This suggests that these factors may have an influence on rGS and GS levels. The trend of increasing numbers of no SUI patients from Q1 to Q4 may suggest that higher rGS and GS levels are associated with a lower risk of SUI. The decreasing numbers of SUI patients from Q1 to Q4 also support this observation (Table 2 and Supplementary Table 1).

**Table 2:** Clinical characteristics of the patients according to the relative grip strength before PSM

**Characteristic**	**Relative grip strength**				***P value***
	**Q1**	**Q2**	**Q3**	**Q4**	
Total patients	1065	1066	1066	1066	
Age, y					< 0.001
Mean, SD	58.8, 16.6	51.7, 16.8	45.7, 16.3	39.4, 14.0	< 0.001
< 40	157 (14.7)	293 (27.5)	430 (40.3)	585 (54.9)	
≥ 40	908 (85.3)	773 (72.5)	636 (59.7)	481 (45.1)	
Race					< 0.001
Non-Hispanic white	497 (46.7)	417 (39.1)	426 (40.0)	482 (45.2)	
Non-Hispanic black	235 (22.1)	242 (22.7)	272 (25.5)	226 (21.2)	
Mexican American	145 (13.6)	152 (14.3)	106 (9.9)	65 (6.1)	
Other Hispanic	123 (11.5)	125 (11.7)	102 (9.6)	69 (6.5)	
Other	65 (6.1)	130 (12.2)	160 (15.0)	224 (21.0)	
Marital status					0.001
Married	435 (40.8)	512 (48.0)	491 (46.1)	523 (49.1)	
Unmarried	630 (59.2)	554 (52.0)	575 (53.9)	543 (50.9)	
Education					< 0.001
Less than high school	294 (27.6)	235 (22.0)	177 (16.6)	108 (10.1)	
High school or equivalent	277 (26.0)	234 (22.0)	187 (17.5)	174 (16.3)	
College or above	494 (46.4)	597 (56.0)	702 (65.9)	784 (73.5)	
Body mass index, kg/m^2^					< 0.001
Mean, SD	36.49, 8.66	31.12, 5.81	27.36, 4.67	23.36, 3.64	< 0.001
Normal (< 25,0)	65 (6.1)	148 (13.9)	353 (33.1)	755 (70.8)	
Overweight (250–29.9)	168 (15.8)	323 (30.3)	431 (40.4)	255 (23.9)	
Obese (≥ 30.0)	832 (78.1)	595 (55.8)	282 (26.5)	56 (5.3)	
Hypertension					< 0.001
No	659 (61.9)	434 (40.7)	317 (29.7)	171 (16.0)	
Yes	406 (38.1)	632 (59.3)	749 (70.3)	895 (84.0)	
Diabetes mellitus					< 0.001
Yes	271 (25.4)	142 (13.3)	73 (6.8)	24 (2.3)	
No	746 (70.0)	889 (83.4)	970 (91.0)	1033 (96.9)	
Borderline	48 (4.5)	35 (3.3)	23 (2.2)	9 (0.8)	
Smoking status					< 0.001
Never	661 (62.1)	695 (65.2)	710 (66.6)	706 (66.2)	
Former	257 (24.1)	208 (19.5)	160 (15.0)	156 (14.6)	
Current	147 (13.8)	163 (15.3)	196 (18.4)	204 (19.1)	
Physical activity status					
Vigorous					< 0.001
Yes	71 (6.7)	147 (13.8)	222 (20.8)	351 (32.9)	
No	994 (93.3)	919 (86.2)	844 (79.2)	715 (67.1)	
Moderate					< 0.001
Yes	337 (31.6)	462 (43.3)	502 (47.1)	541 (50.8)	
No	728 (68.4)	604 (56.7)	564 (52.9)	525 (49.2)	
SUI					< 0.001
No SUI	602 (56.5)	743 (69.7)	828 (77.7)	912 (85.6)	
SUI	463 (43.5)	323 (30.3)	238 (22.3)	154 (14.4)	
Monthly SUI	303 (28.5)	235 (22.0)	195 (18.3)	137 (12.9)	
Weekly SUI	160 (15.0)	88 (8.3)	43 (4.0)	17 (1.6)	
Blood urea nitrogen (mmol/L)	14.72, 7.80	12.51, 5.31	11.36, 4.78	11.07, 3.85	< 0.001
Creatinine (mg/dL)	0.90, 0.72	0.77, 0.28	0.75, 0.25	0.76, 0.22	< 0.001
Uric acid (mg/dL)	5.52, 1.51	5.04, 1.24	4.67, 1.11	4.33, 1.01	< 0.001

The total rGS levels of the quartiles in the study population were: 0.414–1.552 kg (Q1), 1.552–1.933 kg (Q2), 1.935–2.349 kg (Q3), and 2.352–4.041 kg (Q4)

*Abbreviations*: *PSM* Propensity score matching, *SUI* Stress urinary incontinence, *Q1-Q4* Quartile1-Quartile4

It seems that the differences in various demographic and clinical characteristics between the four groups based on rGS and GS quartiles are statistically significant, indicating that these factors may have an impact on the development and severity of SUI. Further analysis and adjustments for these variables may be necessary to better understand the relationship between rGS, GS, and SUI.

### Association of GS or rGS and prevalence or severity risk of SUI before PSM

**Fig. 3:**
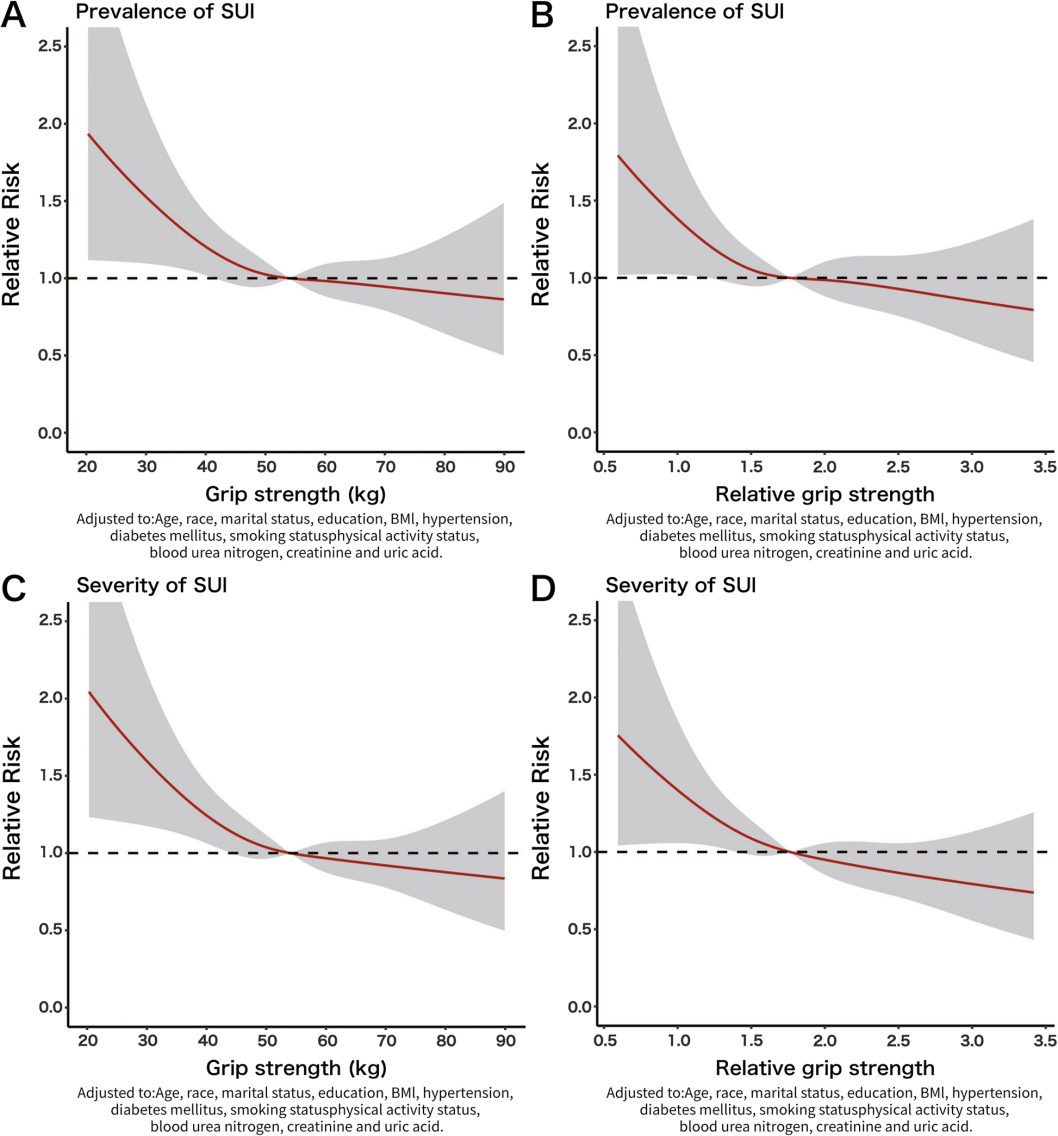
Association of GS or rGS and prevalence or severity risk of SUI before PSM

Logistic regression models were conducted to identify the relationship between levels of GS or rGS and prevalence or severity risk of SUI, based on the quartiles of GS and rGS distribution (Q1-Q4). The results showed that in all models, the risks of prevalence and severity of SUI decreased along with the increasing levels of GS and rGS, as shown in Fig. 3. As indicated in Table 3, in all models, GS and rGS were found to be independent risk factors for SUI.

**Table 3:** Adjusted odds ratios for associations between the grip strength, relative grip strength and prevalence or severity risk of SUI in NHANES 2011–2014 before PSM

**Characteristic**	**Model 0**		**Model 1**		**Model 2**		**Model 3**		**Model 4**	
	**aOR (95% CI)**	***P* value**	**aOR (95% CI)**	***P* value**	**aOR (95% CI)**	***P* value**	**aOR (95% CI)**	***P* value**	**aOR (95% CI)**	***P* value**
**Prevalence of SUI**										
Grip strength (kg)		** < 0.001**		** < 0.001**		** < 0.001**		** < 0.001**		** < 0.001**
Q1	Reference		Reference		Reference		Reference		Reference	
Q2	**0.609 (0.506–0.732)**	** < 0.001**	**0.669 (0.553–0.810)**	** < 0.001**	**0.680 (0.561–0.825)**	** < 0.001**	**0.716 (0.589–0.871)**	**0.001**	**0.729 (0.599–0.888)**	**0.002**
Q3	**0.545 (0.453–0.657)**	** < 0.001**	**0.637 (0.524–0.774)**	** < 0.001**	**0.624 (0.512–0.761)**	** < 0.001**	**0.681 (0.556–0.834)**	** < 0.001**	**0.701 (0.572–0.859)**	**0.001**
Q4	**0.472 (0.390–0.571)**	** < 0.001**	**0.576 (0.469–0.708)**	** < 0.001**	**0.546 (0.441–0.677)**	** < 0.001**	**0.610 (0.490–0.759)**	** < 0.001**	**0.633 (0.508–0.789)**	** < 0.001**
Relative grip strength		** < 0.001**		** < 0.001**		** < 0.001**		** < 0.001**		** < 0.001**
Q1	Reference		Reference		Reference		Reference		Reference	
Q2	**0.565 (0.473–0.675)**	** < 0.001**	**0.621 (0.518–0.744)**	** < 0.001**	**0.650 (0.541–0.780)**	** < 0.001**	**0.699 (0.580–0.842)**	** < 0.001**	**0.692 (0.575–0.832)**	** < 0.001**
Q3	**0.374 (0.310–0.451)**	** < 0.001**	**0.453 (0.373–0.549)**	** < 0.001**	**0.465 (0.382–0.565)**	** < 0.001**	**0.521 (0.426–0.638)**	** < 0.001**	**0.517 (0.423–0.632)**	** < 0.001**
Q4	**0.220 (0.178–0.271)**	** < 0.001**	**0.299 (0.240–0.371)**	** < 0.001**	**0.312 (0.250–0.390)**	** < 0.001**	**0.364 (0.289–0.459)**	** < 0.001**	**0.365 (0.290–0.459)**	** < 0.001**
**Severity of SUI**										
Grip strength (kg)		** < 0.001**		** < 0.001**		** < 0.001**		** < 0.001**		** < 0.001**
Q1	Reference		Reference		Reference		Reference		Reference	
Q2	**0.593 (0.495–0.711)**	** < 0.001**	**0.652 (0.541–0.786)**	** < 0.001**	**0.678 (0.562–0.819)**	** < 0.001**	**0.710 (0.587–0.859)**	** < 0.001**	**0.727 (0.600–0.881)**	**0.001**
Q3	**0.523 (0.435–0.628)**	** < 0.001**	**0.604 (0.500–0.731)**	** < 0.001**	**0.616 (0.507–0.747)**	** < 0.001**	**0.651 (0.535–0.791)**	** < 0.001**	**0.678 (0.555–0.829)**	** < 0.001**
Q4	**0.447 (0.370–0.540)**	** < 0.001**	**0.542 (0.443–0.663)**	** < 0.001**	**0.549 (0.446–0.675)**	** < 0.001**	**0.586 (0.475–0.722)**	** < 0.001**	**0.623 (0.502–0.772)**	** < 0.001**
Relative grip strength		** < 0.001**		** < 0.001**		** < 0.001**		** < 0.001**		** < 0.001**
Q1	Reference		Reference		Reference		Reference		Reference	
Q2	**0.549 (0.461–0.653)**	** < 0.001**	**0.616 (0.515–0.737)**	** < 0.001**	**0.649(0.542–0.778)**	** < 0.001**	**0.681 (0.567–0.818)**	** < 0.001**	**0.697 (0.580–0.839)**	** < 0.001**
Q3	**0.355 (0.295–0.427)**	** < 0.001**	**0.451 (0.366–0.555)**	** < 0.001**	**0.464 (0.376–0.573)**	** < 0.001**	**0.494 (0.400–0.611)**	** < 0.001**	**0.514 (0.414–0.639)**	** < 0.001**
Q4	**0.208 (0.169–0.256)**	** < 0.001**	**0.309 (0.238–0.401)**	** < 0.001**	**0.321 (0.247–0.419)**	** < 0.001**	**0.348 (0.266–0.455)**	** < 0.001**	**0.371 (0.282–0.488)**	** < 0.001**

Adjusted covariates: Model 0: univariate analysis; Model 1: age and BMI; Model 2: model 1 variables plus race, education and marital status; Model 3: model 2 variables plus hypertension and diabetes mellitus; Model 4: model 3 variables plus smoking status, physical activity status, blood urea nitrogen, creatinine and uric acid

*Abbreviations*: *PSM* Propensity score matching, *SUI* Stress urinary incontinence, *BMI* Body mass index, *CI* Confidence interval, *aOR* Adjusted odds ratio, *PSM* Propensity score matching, *Q1-Q4* Quartile1-Quartile4: The total GS levels of the quartiles in the study population were: 15.9–47.7 kg (Q1), 47.8–55.4 kg (Q2), 55.5–63.1 kg (Q3), and 63.2–104.4 kg (Q4). The total rGS levels of the quartiles in the study population were: 0.414–1.552 kg (Q1), 1.552–1.933 kg (Q2), 1.935–2.349 kg (Q3), and 2.352–4.041 kg (Q4)

Specifically, in model 4, the comparison results of prevalence risk of SUI demonstrated that for GS, Q2 vs. Q1, the adjusted odds ratio (aOR) was 0.729 (95%CI = 0.599–0.888, *p* = 0.002); Q3 vs. Q1, the adjusted odds ratio (aOR) was 0.701 (95%CI = 0.572–0.859, *p* = 0.001); Q4 vs. Q1, the adjusted odds ratio (aOR) was 0.633 (95%CI = 0.508–0.789, *p* < 0.001); for rGS, Q2 vs. Q1, the adjusted odds ratio (aOR) was 0.692 (95%CI = 0.575–0.832, *p* < 0.001); Q3 vs. Q1, the adjusted odds ratio (aOR) was 0.517 (95%CI = 0.423–0.632, *p* < 0.001); Q4 vs. Q1, the aOR was 0.365 (95%CI = 0.290–0.459, *p* < 0.001). Similarly, for severity risk of SUI, for GS, Q4 vs. Q1, the aOR was 0.727 (95%CI = 0.600–0.881, *p* = 0.001), while for rGS, Q4 vs. Q1, the aOR was 0.371 (95%CI = 0.282–0.488, *p* < 0.001).

### Identification of influence factors of SUI after PSM

**Fig. 4:**
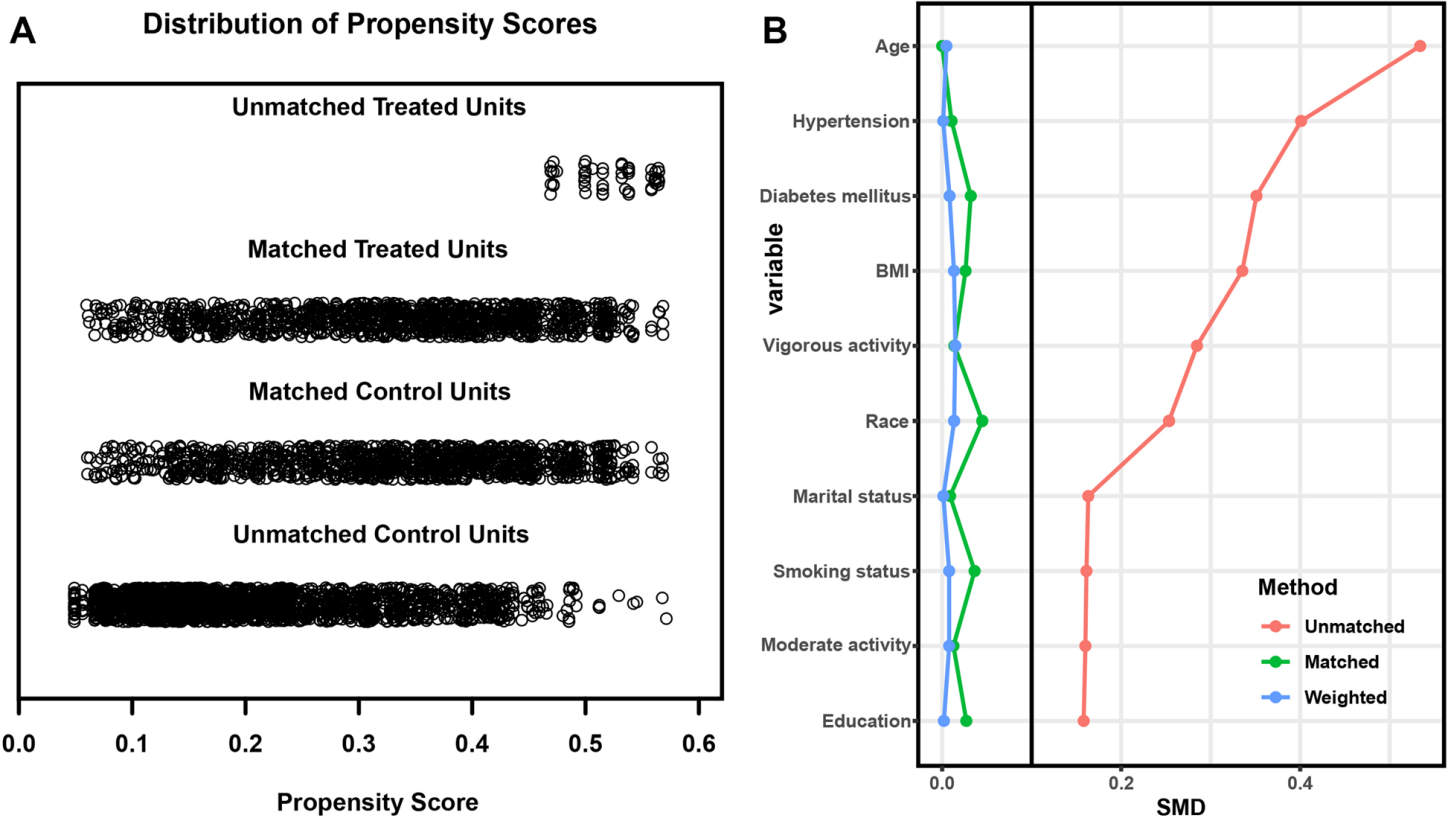
Distribution of propensity scores of 1:1 PSM

**Fig. 5:**
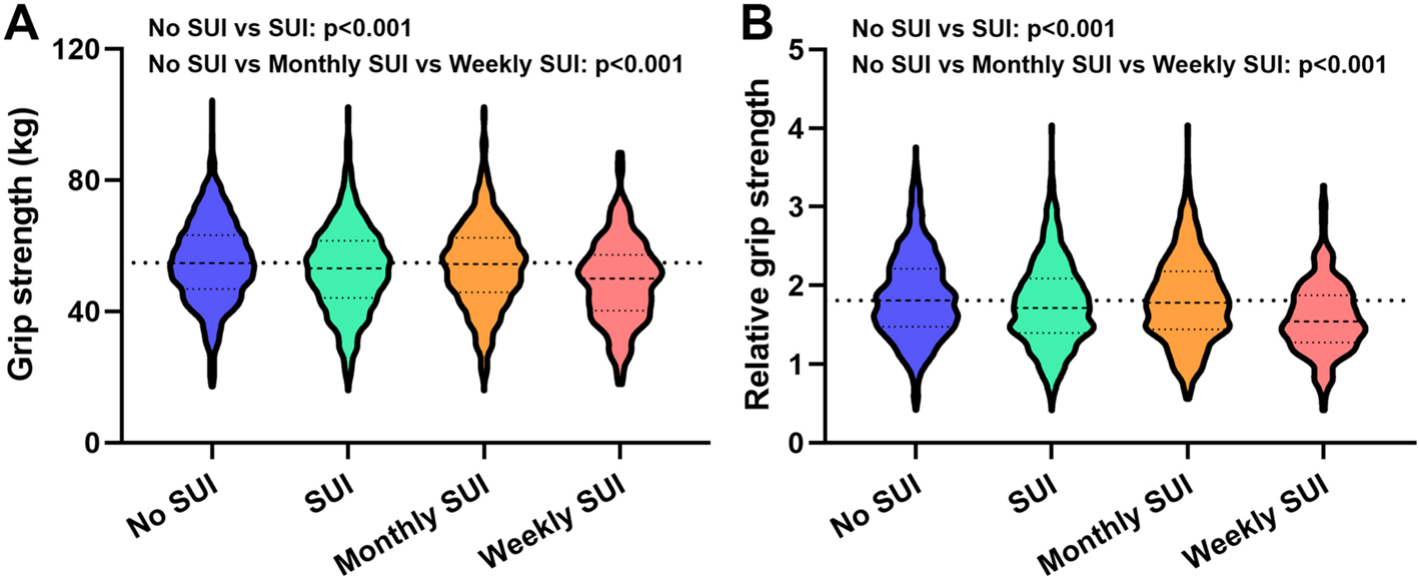
GS and rGS levels of no SUI, SUI, monthly SUI and weekly SUI patients after PSM

Then we performed PSM to adjust the potential influence of other variables. As shown in Fig. 4 and Figure S1, age, race, marital status, education, BMI, diabetes mellitus, smoking status, physical activity status, blood urea nitrogen, creatinine and uric acid were of significant heterogeneity before PSM. After conducting a 1:1 PSM, the propensity score of the matched variables tended to be uniformed. A total of 2280 patients were included, and their clinical characteristics were analyzed with regard to the presence or absence of SUI, as presented in Supplementary Table 2. Notably, a significant difference was observed between the two groups in terms of GS and rGS levels (*p* < 0.001). Specifically, patients without SUI had higher mean levels of GS (55.09, 12.38) and rGS (1.86, 0.54) compared to those with SUI [(52.87, 13.02) for mean GS; (1.76, 0.54) for rGS]. Figure 5 illustrates the GS and rGS levels of different groups after PSM, indicating that SUI patients had lower GS and rGS levels than those without SUI (*p* < 0.001).

### Association of GS or rGS and prevalence or severity risk of SUI after PSM

**Fig. 6:**
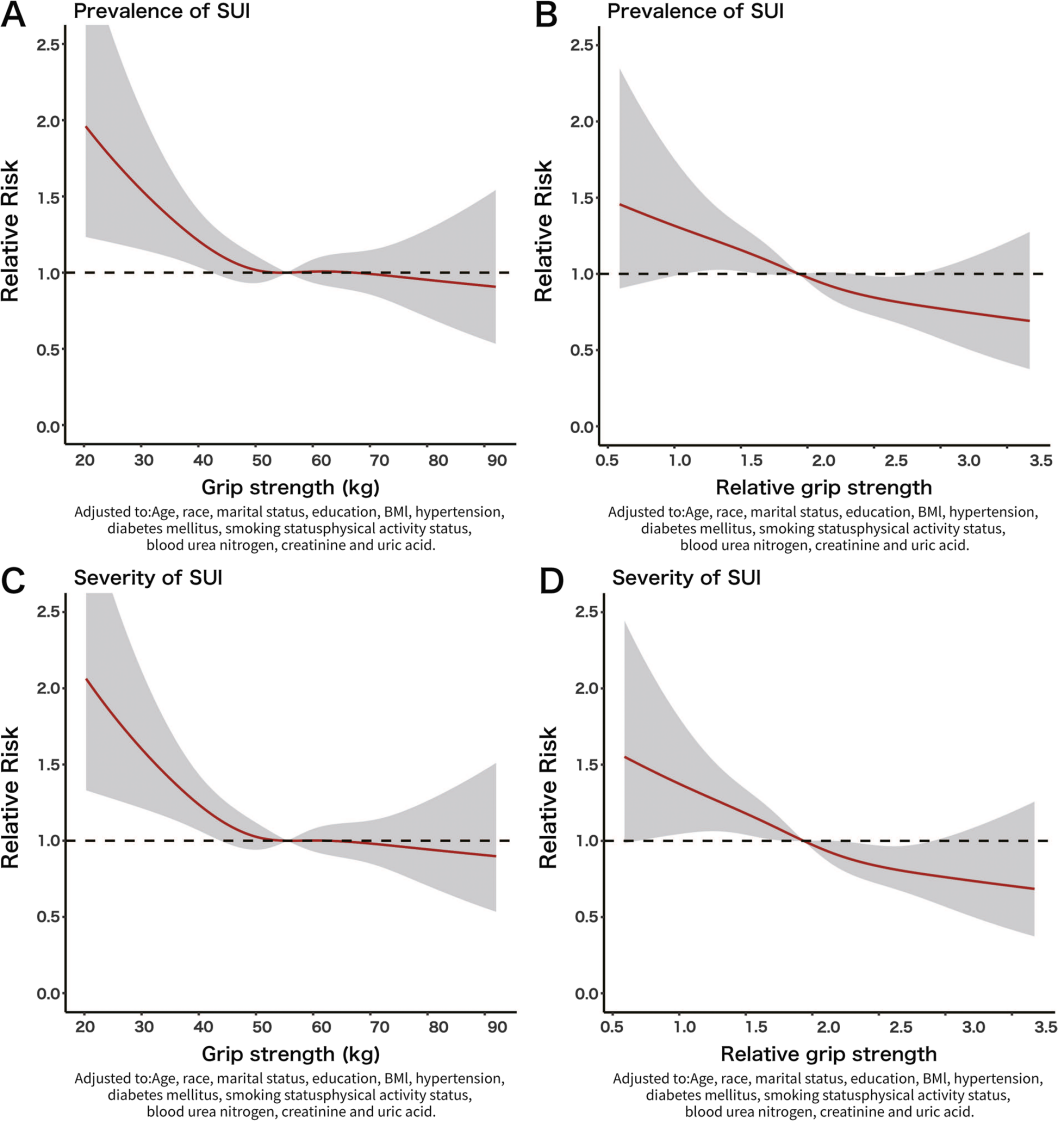
Association of GS or rGS and prevalence or severity risk of SUI after PSM

The study utilized logistic regression models to investigate the potential relationship between GS or rGS and the prevalence or severity risk of SUI after PSM. By controlling for potential bias through PSM, the study found that rGS still demonstrated a stronger association with the prevalence or severity risk of SUI compared to GS. Specifically, in model 4, the prevalence risk of SUI was significantly lower for patients with higher rGS levels (Q4 vs. Q1: aOR = 0.439, 95%CI = 0.326–0.592, *p* < 0.001) compared to those with lower levels. Similar findings were observed for severity risk of SUI. The results from Fig. 6 show a significant inverse relationship between the levels of GS and rGS and the prevalence and severity risk of SUI (*p* < 0.001). These findings are summarized in Table 4.

**Table 4:** Adjusted odds ratios for associations between the grip strength, relative grip strength and prevalence or severity risk of SUI in NHANES 2011–2014 after PSM

**Characteristic**	**Model 0**		**Model 1**		**Model 2**		**Model 3**		**Model 4**	
	**aOR (95% CI)**	***P* value**	**aOR (95% CI)**	***P* value**	**aOR (95% CI)**	***P* value**	**aOR (95% CI)**	***P* value**	**aOR (95% CI)**	***P* value**
**Prevalence of SUI**										
Grip strength (kg)		**0.002**		0.002		0.002		0.002		0.002
Q1	Reference		Reference		Reference		Reference		Reference	
Q2	**0.764 (0.611–0.956)**	**0.019**	**0.764 (0.611–0.956)**	**0.019**	**0.764 (0.611–0.956)**	**0.019**	**0.764 (0.611–0.956)**	**0.019**	**0.764 (0.611–0.956)**	**0.019**
Q3	**0.788 (0.627–0.991)**	**0.041**	**0.788 (0.627–0.991)**	**0.041**	**0.788 (0.627–0.991)**	**0.041**	**0.788 (0.627–0.991)**	**0.041**	**0.788 (0.627–0.991)**	**0.041**
Q4	**0.646 (0.514–0.814)**	** < 0.001**	**0.646 (0.514–0.813)**	** < 0.001**	**0.646 (0.514–0.813)**	** < 0.001**	**0.646 (0.514–0.813)**	** < 0.001**	**0.646 (0.514–0.813)**	** < 0.001**
Relative grip strength		** < 0.001**		** < 0.001**		** < 0.001**		** < 0.001**		** < 0.001**
Q1	Reference		Reference		Reference		Reference		Reference	
Q2	**0.727 (0.590–0.895)**	**0.003**	**0.685 (0.554–0.847)**	** < 0.001**	**0.685 (0.554–0.847)**	** < 0.001**	**0.685 (0.554–0.847)**	** < 0.001**	**0.685 (0.554–0.847)**	** < 0.001**
Q3	**0.760 (0.605–0.954)**	**0.018**	**0.657 (0.515–0.838)**	**0.001**	**0.657 (0.515–0.838)**	**0.001**	**0.657 (0.515–0.838)**	**0.001**	**0.657 (0.515–0.838)**	**0.001**
Q4	**0.589 (0.458–0.758)**	** < 0.001**	**0.439 (0.326–0.592)**	** < 0.001**	**0.439 (0.326–0.592)**	** < 0.001**	**0.439 (0.326–0.592)**	** < 0.001**	**0.439 (0.326–0.592)**	** < 0.001**
**Severity of SUI**										
Grip strength (kg)										
Q1	Reference		Reference		Reference		Reference		Reference	
Q2	**0.731 (0.591–0.904)**	** < 0.001**	**0.728 (0.588–0.902)**	**0.004**	**0.736 (0.593–0.913)**	**0.005**	**0.742 (0.597–0.923)**	**0.007**	**0.731 (0.586–0.911)**	**0.005**
Q3	**0.715 (0.575–0.889)**	**0.002**	**0.711 (0.570–0.888)**	**0.003**	**0.726 (0.580–0.908)**	**0.005**	**0.732 (0.584–0.918)**	**0.007**	**0.728 (0.579–0.918)**	**0.007**
Q4	**0.573 (0.460–0.715)**	**0.004**	**0.568 (0.450–0.717)**	** < 0.001**	**0.583 (0.460–0.739)**	** < 0.001**	**0.589 (0.463–0.748)**	** < 0.001**	**0.592 (0.463–0.757)**	** < 0.001**
Relative grip strength										
Q1	Reference		Reference		Reference		Reference		Reference	
Q2	**0.688 (0.564–0.839)**	** < 0.001**	**0.641 (0.524–0.737)**	** < 0.001**	**0.645 (0.526–0.790)**	** < 0.001**	**0.645 (0.526–0.792)**	** < 0.001**	**0.641 (0.521–0.790)**	** < 0.001**
Q3	**0.669 (0.538–0.831)**	** < 0.001**	**0.559 (0.442–0.707)**	** < 0.001**	**0.564 (0.445–0.715)**	** < 0.001**	**0.564 (0.444–0.717)**	** < 0.001**	**0.566 (0.443–0.723)**	** < 0.001**
Q4	**0.507 (0.397–0.647)**	** < 0.001**	**0.357 (0.264–0.481)**	** < 0.001**	**0.364 (0.269–0.492)**	** < 0.001**	**0.364 (0.268–0.494)**	** < 0.001**	**0.361 (0.264–0.493)**	** < 0.001**

Adjusted covariates: Model 0: univariate analysis; Model 1: age and BMI; Model 2: model 1 variables plus race, education and marital status; Model 3: model 2 variables plus hypertension and diabetes mellitus; Model 4: model 3 variables plus smoking status, physical activity status, blood urea nitrogen, creatinine and uric acid

*Abbreviations: PSM* Propensity score matching, *SUI* Stress urinary incontinence, *BMI* Body mass index, *CI* Confidence interval, *aOR* Adjusted odds ratio, *PSM* Propensity score matching, *Q1-Q4*, Quartile1-Quartile4: The total GS levels of the quartiles in the study population were: 15.9–47.7 kg (Q1), 47.8–55.4 kg (Q2), 55.5–63.1 kg (Q3), and 63.2–104.4 kg (Q4). The total rGS levels of the quartiles in the study population were: 0.414–1.552 kg (Q1), 1.552–1.933 kg (Q2), 1.935–2.349 kg (Q3), and 2.352–4.041 kg (Q4)

## Discussion

This population-based study, which included 4263 female patients, aimed to investigate the impact of GS and rGS on the prevalence and severity of SUI. The study revealed a negative association between GS or rGS and the risks of SUI prevalence or severity. This finding is consistent with previous reports, which suggest that muscle strength plays a functional and metabolic role in disease prevention [17]. Notably, rGS exhibited a stronger relationship with SUI prevalence and severity than GS. To ensure the credibility of the results, we employed PSM to eliminate bias and confounding factors, and the research findings after PSM were consistent with those obtained before PSM.

Urinary incontinence (UI) is a condition that becomes more prevalent with age and has significant adverse effects on quality of life, particularly in women [18]. The worldwide prevalence of UI ranges from 5 to 70%, with rates as high as 44–57% in middle-aged and postmenopausal women [19, 20]. Stress urinary incontinence (SUI) is the most common subtype of UI, with a prevalence of 45.9% among adult women in the United States [21]. Intrinsic sphincter insufficiency is a primary pathophysiological mechanism of SUI, which is usually related to lower pelvic floor muscle strength [3, 22]. Various mechanisms have been implicated in the decline of muscle mass and function with aging. For example, interleukin-6 and other cytokines can decrease concentrations of growth hormone and insulin-like growth factor-1, stimulate muscle cell loss, and lead to muscle weakness [23]. Additionally, sex hormones decline with aging, resulting in a decrease in muscle mass [24]. The pelvic floor muscles, which include the levator ani muscle group, endopelvic fascia, and supporting ligaments, play an essential role in maintaining continence. Weakness in these tissues can prevent the urethra from generating sufficient pressure to resist the increasing pressure in the bladder, resulting in incontinence [25]. Sarcopenia, diagnosed by measuring grip strength (GS), involves the pelvic floor muscles. Based on these mechanisms, sarcopenia can be considered a cause of the declined muscle mass and function of the pelvis and urethra, which further increases the risk of SUI.

A recent study, which included 92 women, revealed a positive correlation between grip strength (GS) levels and pelvic floor muscle strength, suggesting that low GS may serve as a marker for pelvic floor muscle weakness. Moreover, the SUI group showed significantly lower perineometer measurements of GS compared to other subtypes of urinary incontinence, indicating that low GS may have an adverse effect on the risk of SUI [22].

A prospective cohort study by Suskind et al. [13] involving 1475 female samples aged 70 years or older found that changes in GS were associated with changes in SUI frequency over a period of three years. A decline in GS, with or without adjustment for body mass index (BMI), was significantly associated with an increased risk of SUI.

Similarly, Erdogan et al. [14] investigated 802 female urinary incontinence patients and found that SUI was associated with sarcopenia when muscle mass was adjusted by weight independently. Women with sarcopenia had a 1.5 times higher risk of suffering from SUI than typical women. However, this study did not find any significant relationship between low grip strength and SUI.

The current study has several strengths, including the use of standardized methods for data collection, analysis, and measurement, as well as the representativeness of the data. Nonetheless, several limitations must be acknowledged. Firstly, the retrospective nature of the study and the use of a public database may limit the validity and generalizability of the findings. Secondly, the study only included female samples, thus precluding the investigation of potential sex differences in the association between GS and SUI. Furthermore, since the NHANES database only includes noninstitutionalized individuals, the generalizability of the findings to hospitalized populations is uncertain. Future research should consider conducting multicenter prospective clinical trials to better evaluate the effects of GS and rGS on SUI patients.

## Conclusion

Our study revealed that reduced levels of GS and rGS are associated with a higher prevalence and severity of SUI. The consistency of our results was confirmed after performing PSM.

### Supplementary Information

**Additional file 1: Figure S1.** 1:1 PSM of different groups.

**Additional file 2: Supplementary Table 1.** Clinical characteristics of the patients according to the grip strength before PSM.

**Additional file 3: Supplementary Table 2.** Clinical characteristics of the patients according to the with or without SUI after PSM.

## Data Availability

Publicly available datasets were used in this study. These can be found in NHANES at https://www.cdc.gov/nchs/nhanes/index.htm.

## Abbreviations

SUI: Stress urinary incontinence
UI: Urinary incontinence
GS: Grip strength
rGS: Relative grip strength
NHANES: National Health and Nutrition Examination Survey
PSM: Propensity score matching
CI: Confidence interval
aOR: Adjusted odds ratio
BMI: Body mass index

## References

[1] Rosier P, Schaefer W, Lose G (2017). International continence society good urodynamic practices and terms 2016: urodynamics, uroflowmetry, cystometry, and pressure-flow study. Neurourol Urodyn.

[2] Nygaard IE, Heit M (2004). Stress urinary incontinence. Obstet Gynecol.

[3] Goforth J, Langaker M (2016). Urinary Incontinence in women. N C Med J.

[4] Hannestad YS, Rortveit G, Sandvik H (2000). A community-based epidemiological survey of female urinary incontinence: the Norwegian EPINCONT study. Epidemiology of incontinence in the county of nord-trøndelag. J Clin Epidemiol.

[5] Kalejaiye O, Vij M, Drake MJ (2015). Classification of stress urinary incontinence. World J Urol.

[6] Chapple CR, Cruz F, Deffieux X (2017). Consensus statement of the European urology association and the European urogynaecological association on the use of implanted materials for treating pelvic organ prolapse and stress urinary incontinence. Eur Urol.

[7] Stevens PJ, Syddall HE, Patel HP (2012). Is grip strength a good marker of physical performance among community-dwelling older people?. J Nutr Health Aging.

[8] Bohannon RW (2008). Hand-grip dynamometry predicts future outcomes in aging adults. J Geriatr Phys Ther.

[9] Roberts HC, Denison HJ, Martin HJ (2011). A review of the measurement of grip strength in clinical and epidemiological studies: towards a standardised approach. Age Ageing.

[10] Leong DP, Teo KK, Rangarajan S (2015). Prognostic value of grip strength: findings from the Prospective Urban Rural Epidemiology (PURE) study. Lancet.

[11] Cruz-Jentoft AJ, Bahat G, Bauer J (2019). Sarcopenia: revised European consensus on definition and diagnosis. Age Ageing.

[12] Celis-morales CA, Welsh P, Lyall DM (2018). Associations of grip strength with cardiovascular, respiratory, and cancer outcomes and all cause mortality: prospective cohort study of half a million UK Biobank participants. BMJ.

[13] Suskind AM, Cawthon PM, Nakagawa S (2017). Urinary incontinence in older women: the role of body composition and muscle strength: from the health, aging, and body composition study. J Am Geriatr Soc.

[14] Erdogan T, Bahat G, Kilic C (2019). The relationship between sarcopenia and urinary incontinence. Eur Geriatr Med.

[15] Lawman HG, Troiano RP, Perna FM (2016). Associations of relative handgrip strength and cardiovascular disease biomarkers in U.S. adults, 2011–2012. Am J Prev Med.

[16] Xie H, Ruan G, Deng L (2022). Comparison of absolute and relative handgrip strength to predict cancer prognosis: a prospective multicenter cohort study. Clin Nutr.

[17] Wolfe RR (2006). The underappreciated role of muscle in health and disease. Am J Clin Nutr.

[18] Bardsley A (2016). An overview of urinary incontinence. Br J Nurs.

[19] Milsom I, Gyhagen M (2019). The prevalence of urinary incontinence. Climacteric.

[20] Sussman RD, Syan R, Brucker BM (2020). Guideline of guidelines: urinary incontinence in women. BJU Int.

[21] Abufaraj M, Xu T, Cao C (2021). Prevalence and trends in urinary incontinence among women in the United States, 2005–2018. Am J Obstet Gynecol.

[22] Bag Soytas R, Soytas M, Danacioglu YO (2021). Relationship between the types of urinary incontinence, handgrip strength, and pelvic floor muscle strength in adult women. Neurourol Urodyn.

[23] Roubenoff R (2003). Catabolism of aging: is it an inflammatory process?. Curr Opin Clin Nutr Metab Care.

[24] Leifke E, Gorenoi V, Wichers C (2000). Age-related changes of serum sex hormones, insulin-like growth factor-1 and sex-hormone binding globulin levels in men: cross-sectional data from a healthy male cohort. Clin Endocrinol (Oxf).

[25] Grimes WR, Stratton M (2022). Pelvic Floor Dysfunction [M]. StatPearls.

