# Supplementary figures and images for: Association between grip strength and stress urinary incontinence of NHANES 2011–2014
Source: BMC Womens Health. 2023 Oct 3;23:521. doi: 10.1186/s12905-023-02628-1 (PMC10548619; doi:10.1186/s12905-023-02628-1)

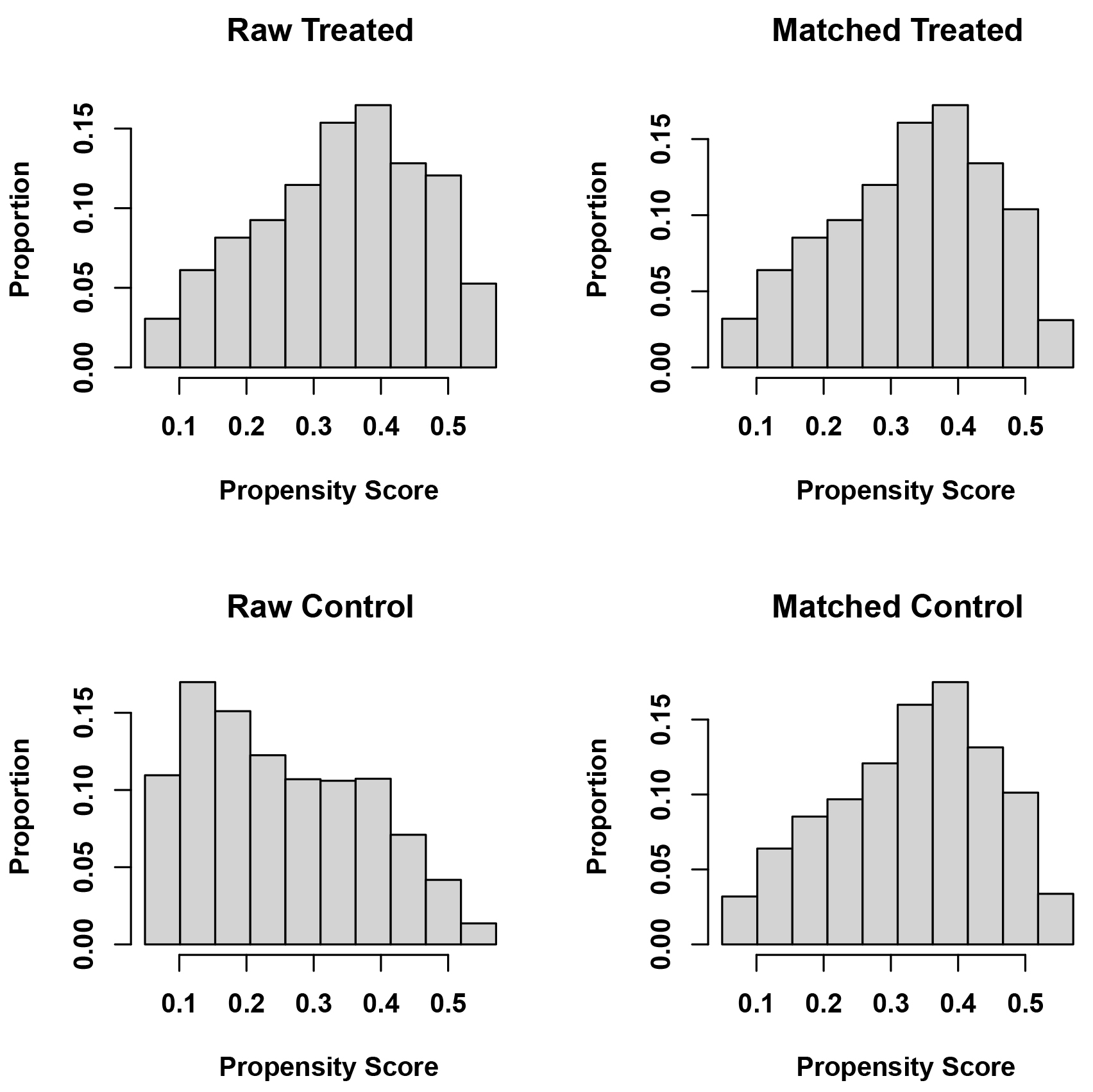

Supplement: Supplementary file 1 — Additional file 1: Figure S1. 1:1 PSM of different groups. [file 12905_2023_2628_MOESM1_ESM.jpg]
