# Supplementary material for: Association between grip strength and stress urinary incontinence of NHANES 2011–2014
Source: BMC Womens Health. 2023 Oct 3;23:521. doi: 10.1186/s12905-023-02628-1 (PMC10548619; doi:10.1186/s12905-023-02628-1)
Supplement: Supplementary file 2 — Additional file 2: Supplementary Table 1. Clinical characteristics of the patients according to the grip strength before PSM. [file 12905_2023_2628_MOESM2_ESM.docx]

**Supplementary Table 1.** Clinical characteristics of the patients according to the grip strength before PSM.

| **Characteristic** | **Grip strength** | | | | **P value** |
| --- | --- | --- | --- | --- | --- |
|  | **Q1** | **Q2** | **Q3** | **Q4** |  |
| Total patients | 1065 | 1058 | 1071 | 1069 |  |
| Age, y |  |  |  |  | <0.001 |
| Mean, SD | 61.2, 17.4 | 49.6, 16.6 | 45.0, 15.5 | 39.7, 12.5 | <0.001 |
| <40 | 158 (14.8) | 328 (31.0) | 419 (39.1) | 560 (52.4) |  |
| ≥40 | 907 (85.2) | 730 (69.0) | 652 (60.9) | 509 (47.6) |  |
| Race |  |  |  |  | <0.001 |
| Non-Hispanic white | 484 (45.4) | 449 (42.4) | 474 (44.3) | 415 (38.8) |  |
| Non-Hispanic black | 141 (13.2) | 169 (16.0) | 258 (24.1) | 407 (38.1) |  |
| Mexican American | 127 (11.9) | 127 (12.0) | 127 (11.9) | 87 (8.1) |  |
| Other Hispanic | 140 (13.1) | 128 (12.1) | 84 (7.8) | 67 (6.3) |  |
| Other | 173 (16.2) | 185 (17.5) | 128 (12.0) | 93 (8.7) |  |
| Marital status |  |  |  |  | 0.021 |
| Married | 452 (42.4) | 514 (48.6) | 511 (47.7) | 484 (45.3) |  |
| Unmarried | 613 (57.6) | 544 (51.4) | 560 (52.3) | 585 (54.7) |  |
| Education |  |  |  |  | <0.001 |
| Less than high school | 301 (28.3) | 219 (20.7) | 168 (15.7) | 126 (11.8) |  |
| High school or equivalent | 243 (22.8) | 207 (19.6) | 218 (20.4) | 204 (19.1) |  |
| College or above | 521 (48.9) | 632 (59.7) | 685 (64.0) | 739 (69.1) |  |
| Body mass index, kg/m^2^ |  |  |  |  | <0.001 |
| Mean, SD | 28.34, 7.07 | 28.88, 7.47 | 29.48, 7.78 | 31.66, 8.07 | <0.001 |
| Normal (<25,0) | 386 (36.2) | 360 (34.0) | 348 (32.5) | 227 (21.2) |  |
| Overweight (250-29.9) | 295 (27.7) | 295 (27.9) | 297 (27.7) | 290 (27.1) |  |
| Obese (≥30.0) | 384 (36.1) | 403 (38.1) | 426 (39.8) | 552 (51.6) |  |
| Hypertension |  |  |  |  | <0.001 |
| No | 568 (53.3) | 374 (35.3) | 329 (30.7) | 310 (29.0) |  |
| Yes | 497 (46.7) | 684 (64.7) | 742 (69.3) | 759 (71.0) |  |
| Diabetes mellitus |  |  |  |  | <0.001 |
| Yes | 214 (20.1) | 129 (12.2) | 96 (9.0) | 71 (6.6) |  |
| No | 815 (76.5) | 891 (84.2) | 954 (89.1) | 978 (91.5) |  |
| Borderline | 36 (3.4) | 38 (3.6) | 21 (2.0) | 20 (1.9) |  |
| Smoking status |  |  |  |  | <0.001 |
| Never | 701 (65.8) | 691 (65.3) | 684 (63.9) | 696 (65.1) |  |
| Former | 236 (22.2) | 188 (17.8) | 198 (18.5) | 159 (14.9) |  |
| Current | 128 (12.0) | 179 (16.9) | 189 (17.6) | 214 (20.0) |  |
| Physical activity status |  |  |  |  |  |
| Vigorous |  |  |  |  | <0.001 |
| Yes | 86 (8.1) | 195 (18.4) | 236 (22.0) | 274 (25.6) |  |
| No | 979 (91.9) | 863 (81.6) | 835 (78.0) | 795 (74.4) |  |
| Moderate |  |  |  |  | <0.001 |
| Yes | 369 (34.6) | 464 (43.9) | 486 (45.4) | 523 (48.9) |  |
| No | 696 (65.4) | 594 (56.1) | 585 (54.6) | 546 (51.1) |  |
| SUI |  |  |  |  | <0.001 |
| No SUI | 667 (62.6) | 776 (73.3) | 808 (75.4) | 834 (78.0) |  |
| SUI | 398 (37.4) | 282 (26.7) | 263 (24.6) | 235 (22.0) |  |
| Monthly SUI | 262 (24.6) | 205 (19.4) | 206 (19.2) | 197 (18.4) |  |
| Weekly SUI | 136 (12.8) | 77 (7.3) | 57(5.3) | 38 (3.6) |  |
| Blood urea nitrogen (mmol/L) | 14.95, 7.79 | 12.35, 4.89 | 11.47, 4.44 | 10.90, 4.58 | <0.001 |
| Creatinine (mg/dL) | 0.88, 0.72 | 0.76, 0.25 | 0.76, 0.27 | 0.79, 0.26 | <0.001 |
| Uric acid (mg/dL) | 5.06, 1.48 | 4.90, 1.32 | 4.81, 1.22 | 4.81, 1.18 | <0.001 |

**Abbreviations:**

PSM, propensity score matching; SUI, stress urinary incontinence; Q1-Q4, Quartile1-Quartile4: The total GS levels of the quartiles in the study population were: 15.9-47.7 kg (Q1), 47.8–55.4 kg (Q2), 55.5–63.1 kg (Q3), and 63.2-104.4 kg (Q4).
