# Supplementary material for: Association between grip strength and stress urinary incontinence of NHANES 2011–2014
Source: BMC Womens Health. 2023 Oct 3;23:521. doi: 10.1186/s12905-023-02628-1 (PMC10548619; doi:10.1186/s12905-023-02628-1)
Supplement: Supplementary file 3 — Additional file 3: Supplementary Table 2. Clinical characteristics of the patients according to the with or without SUI after PSM. [file 12905_2023_2628_MOESM3_ESM.docx]

**Supplementary Table 2.** Clinical characteristics of the patients according to the with or without SUI after PSM.

| **Characteristic** | **All patients** | **No SUI** | **SUI** | **P value** |
| --- | --- | --- | --- | --- |
|  |  | **No. (%)** | **No. (%)** |  |
| Total patients | 2280 | 1140 (50.0) | 1140 (50.0) |  |
| Age, years |  |  |  | 0.745 |
| <40 | 416 (18.2) | 211 (18.5) | 205 (18.0) |  |
| ≥40 | 1864 (81.8) | 929 (81.5) | 935 (82.0) |  |
| Race |  |  |  | 0.643 |
| Non-Hispanic white | 1066 (46.8) | 543 (47.6) | 523 (45.9) |  |
| Non-Hispanic black | 602 (26.4) | 304 (26.7) | 298 (26.1) |  |
| Mexican American | 230 (10.1) | 116 (10.2) | 114 (10.0) |  |
| Other Hispanic | 200 (8.8) | 93 (8.2) | 107 (9.4) |  |
| Other | 182 (8.0) | 84 (7.4) | 98 (8.6) |  |
| Marital status |  |  |  | 0.162 |
| Married | 973 (42.7) | 503 (44.1) | 470 (41.2) |  |
| Unmarried | 1307 (57.3) | 637 (55.9) | 670 (58.8) |  |
| Education |  |  |  | 0.849 |
| Less than high school | 481 (21.1) | 235 (20.6) | 246 (21.6) |  |
| High school or equivalent | 535 (23.5) | 270 (23.7) | 265 (23.2) |  |
| College or above | 1264 (55.4) | 635 (55.7) | 629 (55.2) |  |
| Body mass index, kg/m^2^ |  |  |  | 0.614 |
| Normal (<25,0) | 499 (21.9) | 240 (21.1) | 259 (22.7) |  |
| Overweight (250-29.9) | 596 (26.1) | 299 (26.2) | 297 (26.1) |  |
| Obese (≥30.0) | 1185 (52.0) | 601 (52.7) | 584 (51.2) |  |
| Hypertension |  |  |  | 0.834 |
| No | 1127 (49.4) | 561 (49.2) | 566 (49.6) |  |
| Yes | 1153 (50.6) | 579 (50.8) | 574 (50.4) |  |
| Diabetes mellitus |  |  |  | 0.945 |
| Yes | 407 (17.9) | 201 (17.6) | 206 (18.1) |  |
| No | 1787 (78.4) | 895 (78.5) | 892 (78.2) |  |
| Borderline | 86 (3.8) | 44 (3.9) | 42 (3.7) |  |
| Smoking status |  |  |  | 0.726 |
| Never | 1394 (61.1) | 705 (61.8) | 689 (60.4) |  |
| Former | 495 (21.7) | 240 (21.1) | 255 (22.4) |  |
| Current | 391 (17.1) | 195 (17.1) | 196 (17.2) |  |
| Physical activity status |  |  |  |  |
| Vigorous |  |  |  | 0.843 |
| Yes | 257 (11.3) | 127 (11.1) | 130 (11.4) |  |
| No | 2023 (88.7) | 1013 (88.9) | 1010 (88.6) |  |
| Moderate |  |  |  | 0.829 |
| Yes | 873 (38.3) | 439 (38.5) | 434 (38.1) |  |
| No | 1407 (61.7) | 701 (61.5) | 706 (61.9) |  |
| Grip strength (kg) |  |  |  |  |
| Mean, SD | 53.98, 12.75 | 55.09, 12.38 | 52.87, 13.02 | <0.001 |
| Relative grip strength | 1.81, 0.54 | 1.86, 0.54 | 1.76, 0.54 | <0.001 |
| Blood urea nitrogen (mmol/L) | 13.31, 6.28 | 13.21, 6.56 | 13.42, 5.99 | 0.422 |
| Creatinine (mg/dL) | 0.84, 0.43 | 0.84, 0.47 | 0.83, 0.39 | 0.446 |
| Uric acid (mg/dL) | 5.10, 1.39 | 5.06, 1.36 | 5.15, 1.42 | 0.127 |

**Abbreviations:**

PSM, propensity score matching; SUI, stress urinary incontinence.

For categorical variables, P values were analyzed by chi-square tests. For continuous variables, the t-test for slope was used in generalized linear models.

^a^ Chi-square detected the difference between No SUI group and SUI group.

^b^ Chi-square detected the difference between No SUI group, Monthly SUI group and Weekly SUI group.
